# *In Vitro* Evaluation of *α*-Glucosidase and *α*-Amylase Inhibition in Thai Culinary Vegetables

**DOI:** 10.1155/2024/3625267

**Published:** 2024-09-25

**Authors:** Khakhanang Ratananikom, Vichayut Juntaree, Woragon Wichaiyo, Kris Khunluek, Kantapon Premprayoon, Jittawan Kubola

**Affiliations:** ^1^ Department of Public Health Faculty of Science and Health Technology Kalasin University, Kalasin, Thailand; ^2^ Department of Industrial Engineering Faculty of Engineering and Industrial Technology Kalasin University, Kalasin, Thailand; ^3^ Department of Agricultural Machinery Engineering Faculty of Engineering Rajamangala University of Technology Isan, Khon Kaen Campus, Khon Kaen, Thailand; ^4^ Department of Food Innovation and Processing Faculty of Agricultural Technology Buriram Rajabhat University, Buriram, Thailand

## Abstract

Diabetes management through dietary intervention has gained significant interest, particularly in the use of natural plant-based inhibitors of key enzymes involved in carbohydrate digestion. The objective of this study was to assess the inhibitory effects of ten Thai culinary vegetables on *α*-glucosidase and *α*-amylase, including Chinese chive (*Allium tuberosum*), holy basil (*Ocimum tenuiflorum*), star gooseberry (*Phyllanthus acidus*), galangal (*Alpinia galanga*), bamboo grass (*Tiliacora triandra*), Turkey berry (*Solanum torvum*), cassod tree (*Senna siamea*), dill (*Anethum graveolens* L.), noni (*Morinda citrifolia*), and pearl wattle (*Leucaena leucocephala*). All vegetables were extracted using deionized water at a 1 : 10 ratio in an ultrasonic bath operating at 350 W and a frequency of 50 Hz for 30 minutes. The *α*-glucosidase inhibitory activities of the vegetable extracts ranged from 13.42 ± 0.23% to 79.84 ± 0.47%, while the inhibitory activities against *α*-amylase were relatively modest, ranging from 4.82 ± 3.32% to 27.49 ± 1.67%. Cassod tree exhibited the highest *α*-glucosidase inhibition with the lowest IC_50_ at 126.38 ± 0.98 *μ*g/mL, followed by galangal (203.17 ± 1.05 *μ*g/mL) and holy basil (1,240 ± 20.31 *μ*g/mL), respectively. These results may hold promise for encouraging the consumption of vegetables as a strategy for diabetes management through the targeting of key enzyme inhibition.

## 1. Introduction

As society has continued to develop, the modern way of life has exerted an impact on the health of populations globally. Diabetes mellitus is emerging as a notable health concern, resulting in increased morbidity rates and elevated healthcare expenditures. It is characterized by abnormally high levels of glucose in the bloodstream, known as hyperglycemia, due to the ineffective production of insulin or insufficient responsiveness to it. Such a condition can lead to a range of serious complications in individuals with diabetes, including damage to the blood vessels, kidneys, and eyes, heart attacks, and strokes [1]. By 2030, it has been estimated that a total of 438 million individuals globally are predicted to have diabetes and may be at risk of severe diabetic complications [2].

The effective control and management of hyperglycemia play a pivotal role in diabetes treatment. Contemporary pharmaceutical approaches to address or prevent diabetes have primarily focused on enzyme inhibition. Specifically, *α*-amylase and *α*-glucosidase enzymes are crucial for breaking down complex carbohydrates into simple sugars. Consequently, inhibiting the activity of these enzymes can delay carbohydrate breakdown and glucose absorption [3]. Although, conventional allopathic medications are available, they are costly and have been associated with severe adverse consequences such as discomfort, bloating, flatulence, diarrhea, nausea, and decreased appetite. Moreover, prolonged use of these drugs can lead to drug resistance [1]. Thus, the quest for potent therapeutic agents that can effectively inhibit glycosidase enzymes with minimal side effects has become a challenging endeavor and spurred significant interest in exploring alternative remedies derived from natural sources.

Many epidemiological and empirical studies have shown that plants and herbs produce a wide array of secondary metabolites with the potential to serve as preventive and therapeutic agents, often with reduced toxicity and fewer side effects [4]. Traditionally, various plants and herbs have been used worldwide by local communities for managing diabetes [5]. Current research focuses on evaluating the ability of plants and herbs to inhibit enzymes related to diabetes, particularly *α*-amylase and *α*-glucosidase. Notable examples include black pepper (*Piper guineense*) [3], bell pepper (*Capsicum annuum* L.) [6], Vietnamese mint (*Persicaria odorata*) [7], mango ginger (*Curcuma manga*) [8], red sorrel (*Rumex acetosella*) [9], riceberry (*Oryza sativa*) [10], *Berberis integerrima* Bunge [11], and walnut (*Juglans regia* L.) [12]. While certain plants have accumulated supportive evidence, others continue to lack empirical verification. In addition, identifying potent inhibitors among local plants, herbs, and vegetables can lead to the development of affordable treatment options for communities, especially in developing countries where access to healthcare and medications may be limited. Utilizing local plants for medicinal purposes encourages sustainable practices, as it often involves renewable resources. This approach aligns with global efforts to achieve sustainability in healthcare. Hence, this study aims to assess the inhibitory effects of *α*-amylase and *α*-glucosidase in ten culinary vegetables that are commonly used as main ingredients in Northeastern (Isan) cuisine throughout Thailand. The results could potentially establish vegetables as effective measures for addressing postprandial hyperglycemia, thereby advancing the scientific substantiation of traditional herbal remedies.

## 2. Materials and Methods

### 2.1. Sample Preparation

Ten culinary vegetables that are commonly used as main ingredients in Northeastern cuisine or Isan cuisine in Thailand were studied, namely, Chinese chive, holy basil, star gooseberry, galangal, bamboo grass, Turkey berry, cassod tree, dill, noni, and pearl wattle. Fresh vegetables were procured from three different markets located in Kalasin Province. Upon arrival, three sets of a specific type of vegetable, each sourced from three different markets, were individually cleansed with water to eliminate dirt and other contaminants and then rinsed thoroughly with deionized water. The edible part of each set was then prepared, separated, and homogenized. Approximately 500 g from each of these homogenized samples were combined and rehomogenized to produce a single, uniform composite sample. The characteristics of these vegetables are detailed in Table 1.

### 2.2. Plant Extraction

Samples were extracted using a method described by Ratananikom and Premprayoon with some modifications [13]. Ten grams of freeze-dried vegetables were extracted using deionized water at a 1 : 10 ratio in an ultrasonic bath (LabTech, Korea) at power and frequency of 350 W and 50 Hz, respectively. Extraction time was set at 30 min. Vegetable residue was eliminated by centrifugation. An aliquot of supernatant was recentrifuged before analysis.

### 2.3. *α*-Glucosidase Inhibitory Assay

The inhibitory effects on *α*-glucosidase were assessed using the method described by Kim et al. [14] with minor adjustments. In summary, 50 *µ*L of 10 mg/mL of the vegetable extract was preincubated with 0.05 U/mL *α*-glucosidase in 0.1 M phosphate buffer, pH 6.8 for 10 minutes. Following preincubation, a solution of 1 mM *p*-nitrophenyl-*α*-D-glucopyranoside in 0.1 M phosphate buffer, pH 6.8, was introduced and incubated at 37°C for an additional 30 minutes. To terminate the reaction, 1 mL of 0.1 M sodium carbonate was added. The extent of *α*-glucosidase inhibition was gauged by measuring the absorbance at 405 nm. The percentage of *α*-glucosidase inhibition was calculated using the following equation.

(1) $$\begin{array}{c} \text{Percentage of inhibition}\left(\%\right)=\left[\frac{\left(A-B\right)}{A}\right]\times 100, \end{array}$$

where *A* and *B* were the absorbance values for the control and sample, respectively. To establish a control, an analogous procedure was employed, substituting the vegetable extract with distilled water. The entire experiment was duplicated five times to ensure reliable results.

### 2.4. *α*-Amylase Inhibitory Assay

The inhibitory effect on *α*-amylase was assessed using the method described by Kidane et al. [15] with slight modifications. Concisely, 50 *µ*L of 10 mg/mL of the vegetable extract was preincubated with 0.05 U/mL *α*-amylase in 0.02 M phosphate buffer, pH 6.9, for 10 minutes. Following preincubation, a solution of 1% starch in phosphate buffer, pH 6.9, was introduced and incubated for another 15 minutes. The reaction was terminated by adding 1 mL of the 3,5-dinitrosalicylic acid reagent, after which the mixture was incubated in boiling water for 5 minutes and allowed to cool to room temperature. The degree of *α*-amylase inhibition was assessed by measuring the absorbance at 540 nm. The percentage of *α*-amylase inhibition was calculated using the following equation.

(2) $$\begin{array}{c} \text{Percentage of inhibition}\left(\%\right)=\left[\frac{\left(A-B\right)}{A}\right]\times 100, \end{array}$$

where *A* and *B* were the absorbance values for the control and sample, respectively. To establish a control, an analogous procedure was employed, substituting the vegetable extract with distilled water. The entire experiment was duplicated five times to ensure reliable results.

### 2.5. Determination of the IC_50_ Value

Vegetable extracts demonstrating over 50% inhibition of *α*-glucosidase or *α*-amylase were chosen for IC_50_ evaluation. The IC_50_ value signifies the concentration of vegetable extract necessary to achieve a 50% reduction in *α*-glucosidase or *α*-amylase activity. The IC_50_ value was determined graphically at various concentrations through the relationship between inhibition percentage and concentration according to Kim et al. [14]. The experiments were conducted in triplicate.

### 2.6. Statistical Analysis

The data were presented as mean ± standard deviation. Data conforming to a normal distribution were subjected to one-way analysis of variance (ANOVA), followed by the application of the least significant difference (LSD) test at a significance level of *α* ≤ 0.05.

## 3. Results and Discussion

### 3.1. Screening of *In Vitro α*-Glucosidase and *α*-Amylase Inhibitory Activity

This investigation into the *α*-glucosidase and *α*-amylase inhibitory activities of ten culinary vegetables, integral to Northeastern Thai (Isan) cuisine, uncovered significant variations in their bioactive properties. The studied vegetables, namely, Chinese chive, holy basil, star gooseberry, galangal, bamboo grass, Turkey berry, cassod tree, dill, noni, and pearl wattle, were selected based on their common use in traditional Northeastern Thai dishes. The findings of this study revealed notable differences in their potential to modulate key enzymes involved in carbohydrate digestion, underscoring the intersection of dietary habits and glycemic control.

Using water as a solvent for the extraction of bioactive compounds from vegetables offers several advantages from both practical and scientific perspectives. Water is a nontoxic, nonflammable, and environmentally friendly solvent, making it a safer choice for the environment compared to organic solvents such as methanol or ethanol, which can leave harmful residues in the final extracts. In addition, water is cost-effective and readily available, significantly reducing the expense of large-scale extractions [16]. It is highly efficient at extracting polar compounds, including many bioactive substances such as polyphenols and flavonoids, which are responsible for antioxidant, anti-inflammatory, and enzyme inhibitory activities [17]. Furthermore, a study by Ratananikom et al. [18] found that water is particularly effective in extracting anti-diabetes agents from Thai culinary vegetables, indicating its suitability for such applications.

Among the tested vegetables, the cassod tree (*Senna siamea*) extract exhibited the highest *α*-glucosidase inhibitory activity, with an inhibition percentage of 79.84 ± 0.47. This was followed by galangal (*Alpinia galanga*) and holy basil (*Ocimum tenuiflorum*) with inhibition percentages of 56.89 ± 4.36 and 51.94 ± 0.81, respectively. Notably, these plants showed significant inhibitory activities (*P* < 0.05), suggesting their potential as natural sources for managing postprandial blood glucose levels (Table 2).

Star gooseberry (*Phyllanthus acidus*), bamboo grass (*Tiliacora triandra*), Turkey berry (*Solanum torvum*), dill (*Anethum graveolens* L.), pearl wattle (*Leucaena leucocephala*), and noni (*Morinda citrifolia*) also demonstrated *α*-glucosidase inhibitory activities though to a lesser extent. However, Chinese chives (*Allium tuberosum*) did not exhibit detectable inhibition against *α*-glucosidase under the conditions tested (Table 2).

Dill demonstrated the highest *α*-amylase inhibitory activity with a percentage of 27.49 ± 1.67, significantly different (*P* < 0.05) from other tested vegetables, indicating its potential to slow carbohydrate digestion. Turkey berry also showed noteworthy *α*-amylase inhibition (13.80 ± 0.63%), suggesting its inclusion in the diet could contribute to moderate blood sugar levels. In contrast, holy basil exhibited moderate inhibition (4.82 ± 0.32%), while the remaining vegetables either did not show inhibition or were not detectable under the study conditions.

The inhibition of enzyme activity may be due to the synergistic effect of the chemical constituents present in vegetable extracts. Several factors have been reported to contribute to the differences in *α*-glucosidase and *α*-amylase inhibitory activities, such as the chemical constituents found in vegetables, the origin of the sample, plant genotype, geography, climate, soil fertility, stress level of the plant, type of solvent used, and extraction method [10, 13, 19–22].

The cassod tree emerged as the most potent *α*-glucosidase inhibitor among the tested vegetables, demonstrating an inhibition rate of 79.84 ± 0.47%. This is particularly significant considering the critical role of *α*-glucosidase in the final step of carbohydrate digestion. The inhibition of this enzyme can delay glucose absorption, offering a promising strategy for postprandial blood glucose management. The findings of this study are in line with previous studies that identified flavonoids and other phenolic compounds in cassod trees as potential contributors to inhibitory activity [23, 24]. In addition, a study by Thengyai et al. [23] suggested that buds and heartwoods of cassod trees also showed *α*-glucosidase inhibitory activity. Different levels of anti *α*-glucosidase activity among studies specified different types and quantities of bioactive constituents found from various parts of the cassod tree were used.

With a 56.89 ± 4.36 inhibition percentage, galangal ranks highly in *α*-glucosidase inhibition, coinciding with previous studies by Wongsa et al. [25] and Tian et al. [26]. Interestingly, the flower of galangal was used in this study, while the rhizome [25] and essential oil [26] of galangal were used in the previously mentioned studies. This information indicated that galangal was a good source of *α*-glucosidase inhibitors as a result of various parts of galangal generating *α*-glucosidase inhibitory activities, signifying that galangal could play a significant role in managing diabetes by delaying carbohydrate digestion and absorption, thereby preventing sudden increases in blood sugar levels.

Holy basil presents a noteworthy inhibitory effect on *α*-glucosidase (51.94 ± 0.81%) and a moderate effect on *α*-amylase (4.82 ± 0.32%). The dual inhibitory action suggests that holy basil could be a versatile vegetable for dietary strategies aimed at controlling glucose absorption and postmeal blood sugar levels. The study by Wongsa et al. [25] reported that holy basil did not show inhibitory ability against *α*-amylase, while its inhibition activity against *α*-glucosidase was measured considerably. The data herein agreed with their data in terms of inhibition activity against *α*-glucosidase, though this study did identify some inhibition activity against *α*-amylase of holy basil. This could be explained by the difference in the extraction method. In their study, leaves of holy basil were refluxed in distilled water to obtain the holy basil extract, while this study extracted holy basil leaves by deionized water via ultrasonic extraction. It is feasible that the high temperature used in reflux might have destroyed some bioactive compounds regarding *α*-amylase inhibition.

The study by Ratananikom et al. [18] indicated a high potential in *α*-glucosidase inhibitory and a moderate *α*-amylase inhibitory activity with the IC_50_ value as 96.10 ± 1.64% and 31.81 ± 3.19%, respectively, from the young shoot extract of pearl wattle (*Leucaena leucocephala*). However, a considerable drop of *α*-glucosidase inhibitory and *α*-amylase inhibitory activity was found when using the seed extract of pearl wattle in this study. The *α*-glucosidase inhibitory activity decreased approximately 5 folds to 20.35 ± 0.12%, while *α*-amylase inhibitory activity was undetectable. The marked difference in the inhibitory activity between the extracts could be attributed to the differential phytochemical composition of the young shoots versus the seeds. Plants synthesize a wide array of secondary metabolites, whose concentrations can vary significantly between different parts of the same plant [27]. Young shoots might contain higher levels or different sets of bioactive compounds effective against *α*-glucosidase and *α*-amylase compared to seeds. In addition, the metabolic pathways in plants change as they grow, influenced by developmental stages. This can lead to variations in the types and concentrations of bioactive compounds present [28]. Young shoots, being in an active growth phase, might synthesize certain metabolites at higher levels than seeds, which are more focused on energy storage. This could explain the higher efficacy of young shoot extracts in inhibiting *α*-glucosidase and *α*-amylase activities.

Dill displayed the highest *α*-amylase inhibitory activity (27.49 ± 1.67%), pointing to its unique potential to moderate the breakdown of starch into simpler sugars. The significance of *α*-amylase inhibitors in diabetes management lies in their ability to reduce the rate of starch hydrolysis, thereby blunting postmeal glycemic spikes. The pronounced activity of dill suggests its bioactive compound merit's further investigation in terms of their therapeutic potential, echoing the findings of studies on other rich sources of *α*-amylase inhibitors. Besides being a good source of *α*-amylase inhibition, dill also possessed *α*-glucosidase inhibitory activity with 17.59 ± 1.29%. The current study was the first to uncover the *in vitro* anti-diabetic effects of dill because no significant studies identified them previously [29]. Most reports have focused on *in vivo* anti-diabetic effects, such as the *in vivo* study from Mishra [30], which revealed that the blood glucose level of alloxan-induced diabetic mice decreased significantly after the administration of an aqueous extract from dill seeds. A significant increase in red blood cells, hemoglobin, mean corpuscular hemoglobin, and mean corpuscular hemoglobin content was observed when compared with the control, demonstrating the hypoglycemic and hematopoietic potential of the seed extracts of dill. Furthermore, dill is an abundant source of total phenolic compounds, flavonoids, and antioxidant activities [13, 29].

The inhibitory activities of Turkey berry against *α*-glucosidase and *α*-amylase, as shown in Table 2, offer insightful observations concerning its potential role in the dietary management of blood sugar levels. Turkey berry demonstrated 16.36 ± 1.10% inhibition against *α*-glucosidase and notably higher inhibition at 13.80 ± 0.63% against *α*-amylase. These findings position Turkey berry as a vegetable of interest in the context of diabetes management and metabolic health. Previous studies on its phytochemical composition suggested the presence of various bioactive compounds, including saponins, flavonoids, and alkaloids, which may contribute to its inhibitory activity [31, 32].

Interestingly, most of the vegetable extracts did not inhibit *α*-amylase. This suggests that these vegetables likely lack effective or sufficient quantities of anti-*α*-glucosidase and anti-*α*-amylase compounds. As mentioned earlier, the variety and levels of bioactive compounds present in different plants can vary. However, the absence of detectable inhibitory activity in certain vegetables (e.g., Chinese chives against both enzymes) does not diminish their nutritional value but rather emphasizes the specificity of dietary components in targeting metabolic pathways. This specificity suggests that the selection of dietary interventions for glycemic control can be optimized based on the individual bioactive profiles of foods. However, vegetable extracts exhibiting higher than 50% enzyme inhibition were selected for IC_50_ investigation, i.e., cassod tree, galangal, and holy basil.

### 3.2. IC_50_ of Enzyme Inhibitions

Assessing enzyme inhibition at a fixed concentration yielded relatively limited data [7]. Consequently, the IC_50_ value, which represents the concentration of vegetable extract required to achieve 50% inhibition under specific assay conditions, was utilized in this research to evaluate the effectiveness of the vegetable extract in inhibition of enzymes. However, variations in the IC_50_ value were observed across the different conditions employed among studies [33].

In this study, vegetable extracts demonstrating over 50% inhibition of *α*-glucosidase or *α*-amylase were chosen for further IC_50_ evaluation in order to focus on the most potent inhibitors. This threshold was chosen based on the need to identify the most promising candidates for potential therapeutic application. The decision to focus on extracts with over 50% inhibition aligns with established scientific practices in enzymology and pharmacology, where initial screenings are used to identify lead compounds with substantial bioactivity. This approach is supported by the literature which suggests that higher initial inhibitory percentages are indicative of stronger and more effective enzyme inhibitors, making them prime candidates for detailed kinetic studies and IC_50_ determination. Consequently, this method allows for a more efficient allocation of resources and ensures that the detailed characterization and subsequent research efforts are directed towards the most promising extracts [34, 35].

It was found that the extract of the cassod tree exhibited the most dominant *α*-glucosidase inhibitory activity with an IC_50_ value of 126.38 ± 0.98 *μ*g/mL, while the extracts from galangal and holy basil could inhibit *α*-glucosidase with IC_50_ values of 203.17 ± 1.05 *μ*g/mL and 1,240 ± 20.31 *μ*g/mL, respectively (Table 3). These values provide a quantitative measure of the efficacy of each extract against *α*-glucosidase, with lower IC_50_ values reflecting higher inhibitory potency.

The inhibitory activities of the cassod tree, galangal, and holy basil against *α*-glucosidase, as evidenced by their respective IC_50_ values, highlight the potential of these plants as sources of natural *α*-glucosidase inhibitors. The cassod tree exhibited the most potent inhibitory activity, with an IC_50_ value significantly lower than those of galangal and holy basil. This finding is consistent with previous research indicating the potent bioactivity of *Senna siamea* extracts in managing postprandial glucose levels. Ethanol extract of heartwoods of the cassod tree showed high potency in *α*-glucosidase inhibition with IC_50_ of 14.12 *μ*g/mL [36]. Furthermore, the administration of methanol extract from cassod tree flowers showed a hypoglycemic effect in alloxan-induced diabetic rats [37]. Therefore, consuming these plants that contain bioactive compounds could potentially reduce the incidence of diabetes through the inhibitory pathways of key enzymes. Similarly, the efficacy of galanga in inhibiting *α*-glucosidase corroborates with findings by Tian et al. [26] and Wongsa et al. [25], who identified galangal extracts as promising natural inhibitors for diabetes management.

The implications of this research extend beyond dietary recommendations. The identification of potent *α*-glucosidase and *α*-amylase inhibitors from natural sources such as the vegetables used in Isan cuisine paves the way for the development of functional foods and nutraceuticals aimed at the prevention and management of diabetes. Moreover, these findings contribute to the growing body of evidence supporting the integration of traditional knowledge and modern nutrition science in public health strategies.

## 4. Conclusion

In conclusion, this study highlighted the potential of Thai vegetables from the Northeast, especially cassod tree, galangal, holy basil, Turkey berry, and dill as sources of natural inhibitors for *α*-glucosidase and *α*-amylase, offering promising avenues for the dietary management of blood glucose levels. These findings not only validate the traditional uses of these vegetables in Thai cuisine but also emphasize the role of biodiversity in discovering novel solutions to global health challenges such as diabetes. However, future assays could be employed including *in vivo* studies to assess the efficacy and safety of these vegetable extracts in regulating blood glucose levels in animal models or human trials.

## Figures and Tables

**Table 1:** Characteristics of the selected vegetables.

Scientific name	Common name	Thai name	Used part
*Allium tuberosum*	Chinese chives	Kui chai	Leaves
*Ocimum tenuiflorum*	Holy basil	Kaphrao	Leaves
*Phyllanthus acidus*	Star gooseberry	Mayom	Leaves
*Alpinia galanga*	Galangal	Kha	Flower
*Tiliacora triandra*	Bamboo grass	Yanang	Leaves
*Solanum torvum*	Turkey berry	Makhexpung	Fruit
*Senna siamea*	Cassod tree	Khe lek	Leaves
*Anethum graveolens* L.	Dill	Pak chelao	Leaves
*Morinda citrifolia*	Noni	Yoa	Fruit
*Leucaena leucocephala*	Pearl wattle	Kra thin	Seed

**Table 2:** *α*-Glucosidase and *α*-amylase inhibitory activities of vegetable extracts.

Common name	Percentage of inhibition against	
	*α*-Glucosidase (%)	*α*-Amylase (%)
Chinese chives	Nd	Nd
Holy basil	51.94 ± 0.81^c^	4.82 ± 0.32^c^
Star gooseberry	21.34 ± 1.66^d^	Nd
Galangal	56.89 ± 4.36^b^	Nd
Bamboo grass	17.31 ± 0.46^e^	Nd
Turkey berry	16.36 ± 1.10^e^	13.80 ± 0.63^b^
Cassod tree	79.84 ± 0.47^a^	Nd
Dill	17.59 ± 1.29^e^	27.49 ± 1.67^a^
Noni	13.42 ± 0.23^f^	Nd
Pearl wattle	20.35 ± 0.12^d^	Nd

Remarks: Values were expressed as mean ± standard deviation. Values within each row marked with different superscript lower-case letters indicate significant differences (*P* < 0.05). Nd indicates not detected.

**Table 3:** IC_50_ values against *α*-glucosidase of vegetable extracts.

Common name	IC_50_
Cassod tree	126.38 ± 0.98 *μ*g/mL
Galangal	203.17 ± 1.05 *μ*g/mL
Holy basil	1,240 ± 20.31 *μ*g/mL

Remark: Values were expressed as mean ± standard deviation.

## Data Availability

The data used to support the findings of this study are included within the article materials. Inquiries should be directed to the corresponding author.
